# An Evaluation of a Personalized Multicomponent Commercial Digital Weight Management Program: Single-Arm Behavioral Trial

**DOI:** 10.2196/44955

**Published:** 2023-08-29

**Authors:** Sherry Pagoto, Ran Xu, Tiffany Bullard, Gary D Foster, Richard Bannor, Kaylei Arcangel, Joseph DiVito, Matthew Schroeder, Michelle I Cardel

**Affiliations:** 1 Department of Allied Health Sciences University of Connecticut Storrs, CT United States; 2 WW International, Inc New York, NY United States; 3 Center for Aging Research Regenstrief Institute Indianapolis, IN United States

**Keywords:** weight loss, digital behavioral weight management program, single-arm behavioral trial, personalized weight loss program, ZeroPoint foods, weight management, digital intervention, diet management, exercise

## Abstract

**Background:**

Digital behavioral weight loss programs are scalable and effective, and they provide an opportunity to personalize intervention components. However, more research is needed to test the acceptability and efficacy of personalized digital behavioral weight loss interventions.

**Objective:**

In a 6-month single-arm trial, we examined weight loss, acceptability, and secondary outcomes of a digital commercial weight loss program (WeightWatchers). This digital program included a personalized weight loss program based on sex, age, height, weight, and personal food preferences, as well as synchronous (eg, virtual workshops and individual weekly check-ins) and asynchronous (eg, mobile app and virtual group) elements. In addition to a personalized daily and weekly PersonalPoints target, the program provided users with personalized lists of ≥300 ZeroPoint foods, which are foods that do not need to be weighed, measured, or tracked.

**Methods:**

We conducted a pre-post evaluation of this 6-month, digitally delivered, and personalized WeightWatchers weight management program on weight loss at 3 and 6 months in adults with overweight and obesity. The secondary outcomes included participation, satisfaction, fruit and vegetable intake, physical activity, sleep quality, hunger, food cravings, quality of life, self-compassion, well-being, and behavioral automaticity.

**Results:**

Of the 153 participants, 107 (69.9%) were female, and 65 (42.5%) identified as being from a minoritized racial or ethnic group. Participants’ mean age was 41.09 (SD 13.78) years, and their mean BMI was 31.8 (SD 5.0) kg/m^2^. Participants had an average weight change of −4.25% (SD 3.93%) from baseline to 3 months and −5.05% (SD 5.59%) from baseline to 6 months. At 6 months, the percentages of participants who experienced ≥3%, ≥5%, and ≥10% weight loss were 63.4% (97/153), 51% (78/153), and 14.4% (22/153), respectively. The mean percentage of weeks in which participants engaged in ≥1 aspects of the program was 87.53% (SD 23.40%) at 3 months and 77.67% (SD 28.69%) at 6 months. Retention was high (132/153, 86.3%), and more than two-thirds (94/140, 67.1%) of the participants reported that the program helped them lose weight. Significant improvements were observed in fruit and vegetable intake, physical activity, sleep quality, hunger, food cravings, quality of life, and well-being (all *P* values <.01).

**Conclusions:**

This personalized, digital, and scalable behavioral weight management program resulted in clinically significant weight loss in half (78/153, 51%) of the participants as well as improvements in behavioral and psychosocial outcomes. Future research should compare personalized digital weight loss programs with generic programs on weight loss, participation, and acceptability.

## Introduction

### Background

Digital behavioral weight loss programs are effective [1], and they offer an opportunity for personalization to address the needs of individuals in a scalable manner [2,3]. Personalization can include tailoring intervention characteristics, such as content, timing, or goals [4], and may be based on anthropomorphic data, health behaviors (eg, diet), goals, and psychosocial variables [5]. The conceptual basis of personalized behavioral interventions is that they reduce cognitive load by increasing the relevance of intervention content to each user and eliminating superfluous content, which can then improve intervention acceptability, credibility, and ultimately adherence [6]. Mobile technology has allowed user data to be leveraged to personalize behavioral interventions using algorithms that produce in-the-moment support [6]. Personalizing interventions has generally been shown to improve outcomes in behavior change interventions [4]. Two systematic reviews—one involving 6 interventions and the other involving 31 interventions—have examined the efficacy of personalized technology-based interventions targeting lifestyle behaviors [5,6]. One found that personalized interventions were more effective for weight loss than nonpersonalized or waitlist controls and that participants felt that personalized intervention content was more relevant, helpful, and easier to understand than generic content [5]. Similarly, the other systematic review found that personalized lifestyle interventions have moderate positive effects on lifestyle behaviors, and the authors recommend that future personalized interventions should investigate the integration of multiple types of data from different sources and include personalized features in addition to intervention content [6].

The WeightWatchers (WW) program is a well-validated commercial weight management program that now leverages a personalized eHealth approach [7-10]. WW’s dietary approach assigns each food and drink a points value (ie, PersonalPoints) based on calories, saturated fat, unsaturated fat, added sugar, fiber, and protein [11]. Calories, saturated fat, and added sugar increase the PersonalPoints value of a food, whereas unsaturated fat, fiber, and protein decrease the PersonalPoints value. In addition, foods that represent the cornerstone of a healthy pattern (eg, fruits, vegetables, seafood, lean proteins, and low-fat dairy) are assigned a points value of 0 (ZeroPoint foods [ZPFs]). These ZPFs do not need to be weighed, measured, or tracked. WW’s personalized version of the program, called the PersonalPoints program, creates a fully personalized dietary plan with >28,000 options. Personalization inputs are both *surface level*, including age, height, weight, and sex, and *deep level*, including food preferences, diabetes status, breastfeeding status, physical activity habits, and water consumption. The app uses user-entered data regarding the factors that influence energy needs, including age, height, weight, and sex, to personalize the PersonalPoints budget. This is further personalized based on breastfeeding status and physical activity level, which also directly affect energy needs. Users receive a personalized list of ZPFs that includes foods that form the basis of an overall healthy dietary pattern and can reduce hunger and cravings, given their macronutrient composition (eg, high fiber and high protein). This minimizes hunger because, as ZPFs do not need to be tracked, even if no PersonalPoints remain in a user’s budget for the day, they may eat foods on their ZPF list. The program reinforces physical activity by allowing users to receive additional PersonalPoints for being active. The personalization is also dynamic such that participants can change their food preferences, physical activity habits, nonstarchy vegetable consumption, and water intake at any time, which then changes their PersonalPoints daily and weekly goals as well as their ZPF list (which includes >300 options). The purpose of the PersonalPoints program is to provide dynamic, personalized targets to help members lose weight while improving overall dietary patterns, reducing hunger and cravings, encouraging physical activity, and simplifying diet tracking by not requiring all foods consumed to be tracked.

### Objectives

Our first aim in this 6-month single-arm trial was to examine the effect of this personalized program on percentage weight loss from baseline to 3 and 6 months. Our second aim was to describe participation and acceptability as indicators of program satisfaction. Our third aim was to examine the impact of this program on fruit and vegetable intake, physical activity, sedentary behavior, hunger, and food cravings. Our fourth aim was to examine the impact on sleep, quality of life, self-compassion, overall well-being, and behavioral automaticity at 6 months. This is the first trial to evaluate the WW PersonalPoints program.

## Methods

### Study Design, Settings, and Participants

This study was a 6-month single-arm trial evaluating the effectiveness of a commercially available WW PersonalPoints weight management and wellness program on percentage weight loss at 3 and 6 months. We recruited adults with overweight or obesity who were interested in losing weight by posting recruitment advertisements on Facebook, Instagram, Twitter, Reddit, Craigslist, and Research Match, which connects volunteers to research studies throughout the United States [12,13]. The inclusion criteria for the study were aged 18 to 75 years, BMI between 25 and 45 kg/m^2^, Wi-Fi connectivity at home, having an iPhone (because the app was only available on iOS at the time), English proficiency, self-reported desire to lose weight, and US residence. The exclusion criteria were being pregnant, lactating, or planning to become pregnant during the study period; severe mental illness (eg, bipolar disorder, severe depression, or psychosis); eating disorders; hospitalization for psychiatric disorders during the past 12 months; type 1 or type 2 diabetes; taking medications that affect weight; currently in a structured weight loss program; had bariatric surgery or plans to have any surgery during the study; unable to walk one-fourth of a mile unaided without stopping; smoker or uses nicotine vape daily; medical condition that precludes ability to make dietary changes or increase physical activity; weight loss of ≥5 kg in the last 6 months; WW membership in the last 12 months; major surgery within the previous 6 months; implanted cardiac defibrillator or pacemaker; history of cancer within past 5 years or current treatment for cancer; and unable to attend any virtual workshop meeting times.

Participant screening involved an initial web-based survey, followed by a telephone interview. Eligible participants then completed a web-based baseline survey. Subsequently, they were mailed a WW Conair Bluetooth Scale, and they completed an onboarding process, which included emailed instructions on how to set up the app and Bluetooth scale, including an option to schedule a call with study staff to assist with setup. Study staff checked that all scales were set up before the program commenced. Participants then took part in the 6-month WW PersonalPoints program. Data collection occurred at 3 and 6 months, which involved a web-based survey and a weigh-in on the scale. Participants were compensated with Amazon e-gift cards at baseline (US $20), 3 months (US $50), and 6 months (US $50) and were allowed to keep the Bluetooth scale after the study. The study period was from April 5, 2021 to September 19, 2021. This trial is registered at ClinicalTrials.gov (NCT04302389).

### Ethics Approval

All work was approved by the University of Connecticut Institutional Review Board (institutional review board approval number: H20-0030).

### Intervention

#### Overview

The 6-month intervention included WW’s mobile app (beta version of the PersonalPoints program), weekly group virtual workshops, weekly one-on-one virtual check-ins, and an invitation to a private members-only digital community. The PersonalPoints program guides members toward their weight and wellness goals through a weekly curriculum that is complemented by specific behavioral goals each week across 4 main pillars (food, activity, sleep, and mindset) to drive healthy habits. The WW program is based on recommendations by national and international guidelines to form the foundation for a healthy pattern of eating [14]. Furthermore, the WW app provides members with a self-guided personalized weight management plan that includes a weekly check-in and progress report, which has options to track one’s weight, ask how the week went, and provide an opportunity for reflection, as well as allows for goal setting for the next week. The app also includes food, activity, water, sleep, and weight trackers; meal planning tools; recipes and a food barcode scanner; a personalized ZPFs list; and guided meditations and workouts (Multimedia Appendix 1).

The personalized aspects of the program are as follows. Participants receive a daily PersonalPoints budget based on their age, height, weight, sex, and breastfeeding status. The PersonalPoints budget is dynamic to the extent that these factors not only change, but the participants’ daily PersonalPoints budget also increases when they consume nonstarchy vegetables and water. The personalized dietary plans include >28,000 options, depending on user inputs. Participants also receive a weekly PersonalPoints budget that gives them extra points to use throughout the week. The weekly budget is dynamic, based on changes in age, weight, and the amount of physical activity. Once a member has spent all their daily PersonalPoints, they may spend their weekly PersonalPoints. Up to 4 unused daily PersonalPoints are automatically rolled over into the weekly PersonalPoints budget, but each participant has the option to turn off rollovers to expedite weight loss. Participants also receive a personalized ZPF list based on their food preferences, which they may change at any time. WW has >300 ZPFs. Food preferences are assessed with the questions presented in Multimedia Appendix 2. ZPF lists are balanced with the daily PersonalPoints budget such that a longer ZPF list is accompanied by a smaller daily PersonalPoints budget. Participants also receive a personalized weekly activity target, which represents a modest increase from their current activity level. The questions driving the activity target are described in Multimedia Appendix 2. Participants also receive personalized in-app program content based on their membership type, diabetes status, and tenure in the program.

Participants were asked to complete their profile and log their food intake and exercise daily, weigh in at least once a week but no more than daily, and attend the weekly virtual workshops and individual check-ins. When participants hit a weight loss milestone, they were sent small keychains to commemorate the milestone they achieved (eg, 5 lb, 10 lb, 15 lb, 20 lb, and 25 lb).

#### Virtual Workshops

Virtual workshops were conducted via videoconferencing software and occurred weekly for 6 months. Links to the virtual workshops were emailed weekly to the participants. WW offered meeting times on several different days and times. Study staff assigned each participant a workshop based on their preferred days and times. The workshops, which ranged in size from 16 to 19 participants and lasted between 30 and 60 minutes, were led by a trained WW coach. During the workshops, the coach led a conversation on a topic related to effective weight management and behavior change. The participants also had the opportunity to receive group support by discussing their successes and setbacks as well as help to troubleshoot challenges (Multimedia Appendix 3).

#### Virtual Check-Ins

Virtual check-ins with a WW coach were conducted over Zoom (Zoom Video Communications, Inc) and occurred weekly for 6 months. Participants scheduled their weekly check-ins via Calendly, a web-based appointment scheduling software, after which links to the virtual check-in meetings were emailed to each participant. Check-ins lasted approximately 5 minutes and were conducted one-on-one. During check-ins, coaches queried participants about their progress and setbacks, answered questions, and set goals for the week. Check-ins allowed participants to receive individual support to complement the group support from weekly workshops.

#### Private Members-Only Digital Community

All participants were invited to an optional private members-only digital community group where they could interact with, and support, each other. Participants could share their journey via posts, photographs, videos, and comments. The group also was a place for participants to ask questions as well as to give motivational support to other participants and receive motivational support from them. The group moderator was a WW coach who posted in the group 3 to 4 times per week to share recipes and ask questions to foster conversations among participants regarding healthy lifestyle changes.

#### App Academy

WW offers additional workshops coined the *App Academy* to provide interested participants with a more detailed review of the functionalities and features of the app and to answer participant questions about the app. These workshops are led by a WW coach, last up to 60 minutes, are held twice, and were optional. Of the 153 participants, 27 (17.6%) attended these sessions.

### Measures

#### Weight Change

At baseline, 3 months, and 6 months, weight values were extracted directly from the WW beta app, which synchronized to the WW Bluetooth scale. In rare events where a participant was unable to get their scale to pair with the WW app, participants uploaded a screenshot of their weight through REDCap (Research Electronic Data Capture; Vanderbilt University) using a private link. Participants were asked to step on the scale in the morning, undressed and after voiding. Weight was recorded in pounds directly from the Bluetooth scale mailed to participants. Weight change was defined as the difference between baseline weight and the weight at 3 months or 6 months. Percentage weight change was calculated as weight change divided by baseline weight and multiplied by 100.

#### Participation

Participation was calculated as both the percentage and mean number of weeks out of the 24 total weeks in which participants attended virtual workshops, percentage and mean number of weeks in which they attended weekly wellness check-ins, percentage and mean number of weeks in which they engaged in the private group (ie, posts, comments and replies, and reactions), and percentage and mean number of days in which participants tracked food, physical activity, and weight in the WW app. If a participant tracked at least 1 food in a day, they were given credit for a day of tracking. Given that we offered participants myriad ways to engage, the mean number of weeks in which the participant carried out ≥1 of each of these forms of participation was also calculated.

#### Satisfaction

At the 6-month follow-up, participants were asked 14 questions about how satisfied they were with their experiences in the program. These 14 items were scored using a 5-point Likert scale ranging from 1=strongly disagree to 5=strongly agree. The responses were then dichotomized to strongly disagree, disagree, or neither agree nor disagree (1-3) and agree or strongly agree (4-5).

#### Hunger Visual Analog Scale

At baseline, 3 months, and 6 months, participants rated their hunger over the past week using a slider on a visual analog scale ranging from 0=not at all hungry to 100=extremely hungry.

#### Food Craving Inventory

At baseline, 3 months, and 6 months, participants completed the Food Craving Inventory, which measures food craving via 28 items. In addition to providing a total score (ie, mean of all items), it includes 4 subscales, including high fats (8 items), sweets (8 items), carbohydrates or starches (8 items), and fast-food fats (4 items) rated on a 5-point Likert scale ranging from 1=never to 5=always [15].

#### Fruit and Vegetable Intake

At baseline, 3 months, and 6 months, participants completed the Five-Factor Screener, which contains 19 items that assess the consumption frequency (measured on a scale ranging from 0=never to 5=≥5 times per day) of 21 food groups, including fruits and vegetables. For the analysis, we converted all reported frequencies to daily frequencies based on the Five-Factor Screener scoring guide [16].

#### Global Physical Activity Questionnaire

Physical activity was measured at baseline, 3 months, and 6 months using the Global Physical Activity Questionnaire (GPAQ). Physical activity was categorized as either moderate or vigorous. We defined vigorous (eg, running or lifting heavy loads) and moderate (eg, brisk walking or carrying light loads) physical activities as activities that cause large and small increases in breathing or heart rate for at least 10 minutes continuously, respectively. We asked participants whether (yes or no) they were engaged in vigorous or moderate physical activity, the number of days in a week in which they were engaged in this activity, and the amount of time (in h and min) they spent on a typical day engaging in this activity at work, during travel and during leisure time. We reported total metabolic equivalent (MET) minutes per week for each participant where 1 minute of moderate activity and 1 minute of vigorous activity are equivalent to 4 MET minutes and 8 MET minutes, respectively [17,18]. Sedentary behavior was also measured as the amount of time (in min) spent sitting or reclining on a typical day.

#### Pittsburgh Sleep Quality Index

The Pittsburgh Sleep Quality Index is a 19-item questionnaire that assesses sleep quality and disturbances [18]. It has 7 subscales: sleep duration (1 item), sleep disturbance (9 items), sleep latency (2 items), sleep dysfunction owing to sleepiness (2 items), sleep efficiency (3 items), overall sleep quality (1 item), and need medication to sleep (1 item). The total Pittsburgh Sleep Quality Index score ranges from 0=better sleep to 21=worse sleep [19].

#### Impact of Weight on Quality of Life-Lite Questionnaire

The Impact of Weight on Quality of Life-Lite questionnaire is composed of 31 items rated on a 5-point Likert scale ranging from 1=never true to 5=always true, and it assesses the perception of how weight affects day-to-day life. It has 5 subscales: physical function (11 items), self-esteem (7 items), sexual life (4 items), public distress (5 items), and work (4 items) [20].

#### Self-Compassion Scale

The Self-Compassion Scale is a 26-item measure of self-compassion and consists of 6 subscales: self-kindness, self-judgment, common humanity, isolation, mindfulness, and overidentification. These were scored on a scale ranging from 1=almost never to 5=almost always. A total self-compassion score was obtained by reverse scoring the negative subscale items (self-judgment, isolation, and overidentification) and computing a grand mean of all 6 subscale means [21].

#### World Health Organization-5 Well-Being Index

Well-being was measured at baseline, 3 months, and 6 months using the World Health Organization-5 Well-Being Index, which assesses subjective psychological well-being with 5 items: “I have felt cheerful in good spirits,” “I have felt calm and relaxed,” “I have felt active and vigorous,” “I woke up feeling fresh and rested,” and “My daily life has been filled with things that interest me.” The 5-point Likert scale items range from 0=at no time to 5=all the time and were summed to create a total score [22].

#### Behavioral Automaticity

Behavioral automaticity for 11 behaviors, of which 7 were healthy behaviors and 4 were unhealthy behaviors, was measured at baseline, 3 months, and 6 months using the Self-Report Behavioral Automaticity Index [23]. For each behavior in which the participant indicates that they engage in it anywhere from rarely to always, the participant is asked to respond to 4 items assessing whether the behavior is something “I do automatically,” “I do without having to consciously remember,” “I do without thinking,” and “I start doing it before I realize I’m doing it.” Responses to each item are rated on a 7-point Likert scale ranging from 1=strongly disagree to 7=strongly agree. A mean score was calculated for each behavior; next, mean scores were calculated for the 7 healthy behaviors to produce a positive behavioral automaticity score, and mean scores were calculated for the 4 unhealthy behaviors to produce a negative behavioral automaticity score.

### Sample Size and Data Collection

With α=.05, assuming an SD of 6% based on our previous trial [24] and 15% attrition, a sample of 150 was determined to have 80% power to detect mean 6-month weight change of +1.5% to −1.5% or greater using a 2-tailed, 1-sample *t* test. Our final sample included 153 participants, with 140 (91.5%; attrition: 8.5%) completing the 6-month follow-up.

### Data Analysis

We used REDCap for data collection and monitoring the completion of study assessments. Data analysis was performed using SPSS software for Windows (version 27.0; IBM Corp). We calculated weight change and percentage weight change from baseline to 3 months and baseline to 6 months using the intention-to-treat approach. Given that weight at 3 months and weight at 6 months are likely not missing completely at random (ie, the probabilities of missing weight at 3 months or 6 months are likely to correlate with participants’ baseline weight, sociodemographic characteristics, or other secondary outcomes), we used the multiple imputations approach to impute the missing weight at 3 months and 6 months [25,26]. Specifically, we implemented an iterative Markov chain Monte Carlo method with multivariate normal distribution [27] to impute the missing values of weight at 3 and 6 months, with baseline weight, sociodemographic characteristics, and all other secondary outcomes (at 3 and 6 months) as covariates. We used a 2-tailed paired *t* test to evaluate weight change from baseline to 3 months and from baseline to 6 months. We also calculated the proportion of participants who achieved weight loss of ≥3%, ≥5%, and ≥10%. Using the same analytic approach as examining weight, we assessed changes in hunger, food craving, dietary intake, physical activity, and well-being from baseline to 3 months and from baseline to 6 months. Missing values in these secondary measures were imputed by baseline observation carried forward.

## Results

### Participants

**Figure 1:**
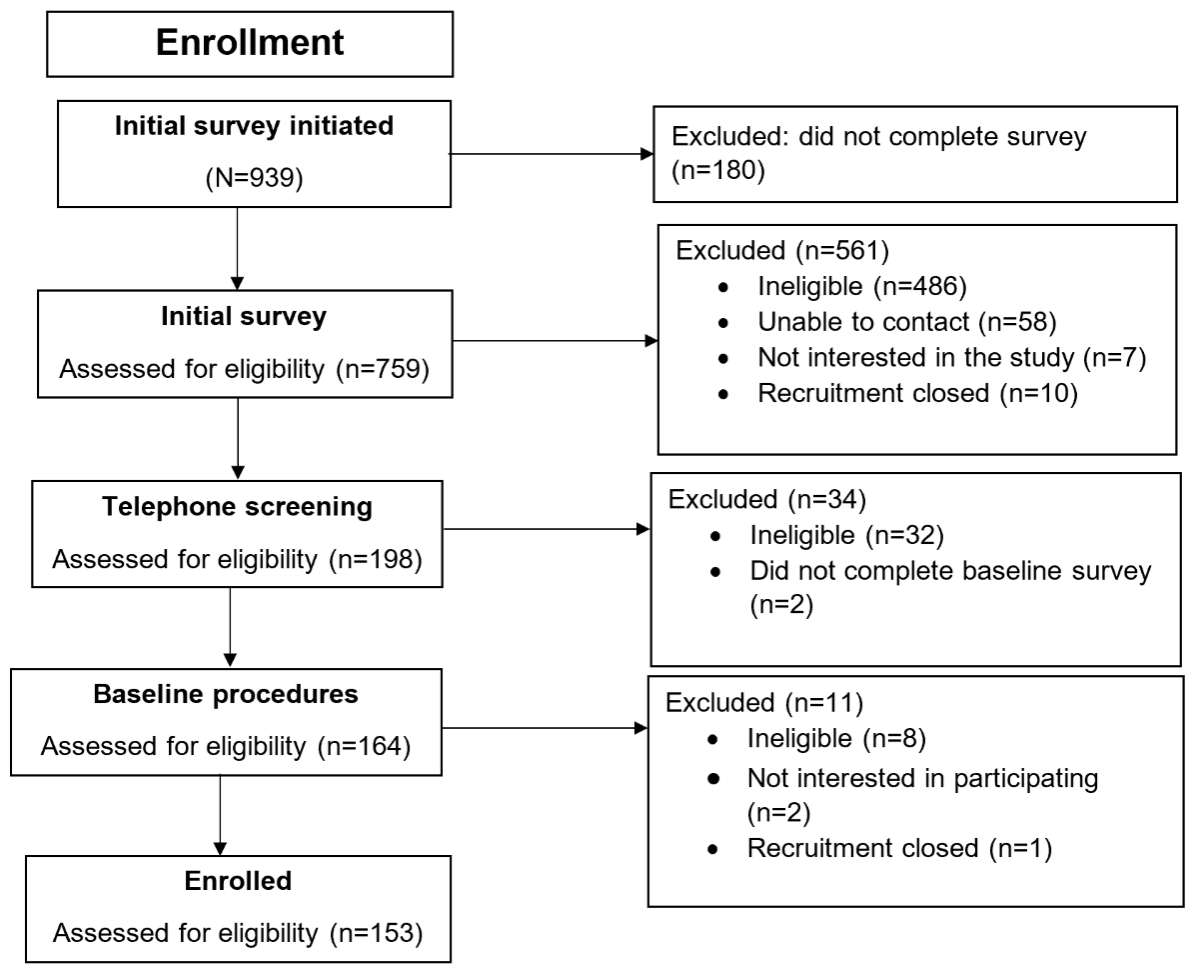
CONSORT (Consolidated Standards of Reporting Trials) diagram for participants screened.

In total, 759 individuals completed the initial screening survey, of whom 153 (20.2%) were enrolled in the study and 606 (79.8%) were excluded from the study (Figure 1). Among these 606 individuals, the most common reasons for exclusion were not having an iPhone (288/606, 47.5%), BMI out of range (88/606, 14.5%), not providing consent (72/606, 11.9%), unable to contact (58/606, 9.6%), and being a WW member in the past 12 months (55/606, 9.1%). Enrolled participants (n=153; n=107, 69.9% female and n=65, 42.5% identifying with a minoritized racial or ethnic background) were on average aged 41.1 (SD 13.8) years and came from 34 states. Most of the participants were married or cohabiting (96/153, 62.7%), were in full-time employment (100/153, 65.4%), and had an annual household income of ≥US $80,000 (98/153, 64.1%; Table 1). Retention, defined as the percentage of participants who provided all follow-up data, was 92.2% (141/153) at 3 months and 86.3% (132/153) at 6 months. The percentages of participants who provided follow-up weight data were 93.5% (143/153) at 3 months and 91.5% (140/153) at 6 months. Our sample of 153 participants used a total of 121 different PersonalPoints plans that varied based on their PersonalPoints budget, activity goals, and ZPF lists.

**Table 1:** Demographic characteristics of participants (n=153).

Demographic characteristics		Values
Age (years), mean (SD)		41.1 (13.8)
**Sex, n (%)**		
	Female	107 (69.9)
	Male	46 (30.1)
**Race and ethnicity, n (%)**		
	Hispanic or Latino of any race	19 (12.4)
	Non-Hispanic Asian	20 (13.1)
	Non-Hispanic Black or African American	16 (10.5)
	Non-Hispanic White	88 (57.5)
	Multiracial (non-Hispanic)	10 (6.5)
**Marital status, n (%)**		
	Married or cohabiting	96 (62.8)
	Single	47 (30.7)
	Separated, divorced, or widowed	10 (6.5)
**Highest educational level, n (%)**		
	High school degree or GED^a^ or equivalent	4 (2.6)
	Trade or technical or some college or associate degree	28 (18.3)
	Bachelor’s degree or some graduate school	50 (32.7)
	Master’s degree or doctoral degree	71 (46.4)
**Employment status, n (%)**		
	Full time	100 (65.3)
	Part time	22 (14.4)
	Student	11 (7.2)
	Unemployed, retired, disabled, or homemaker	15 (9.8)
	Other	5 (3.3)
**Annual household income (US $), n (%)**		
	0-39,999	15 (9.8)
	40,000-79,999	40 (26.1)
	≥80,000	98 (64.1)
Former WW^b^ members n (%)		39 (25.5)

^a^GED: General Educational Development Test.

^b^WW: WeightWatchers.

### Weight Change

Participants lost an average of −8.19 (SD 7.51) lb at 3 months and −9.72 (SD 10.78) lb at 6 months (all *P* values <.001), equivalent to a weight loss of −4.25% (SD 3.93%) at 3 months and a weight loss of −5.05% (SD 5.59%) at 6 months (Table 2). At 6 months, the percentages of participants who had ≥3%, ≥5% and ≥10% weight loss were 63.4% (97/153), 51% (78/153), and 14.4% (22/153), respectively.

**Table 2:** Changes in weight, BMI, hunger, food craving, quality of life, physical activity, sedentary behavior, sleep quality, self-compassion, and well-being measured using a 2-tailed sample *t* test (n=153).

Variable				Baseline, mean (SD)		3 months, mean (SD)		3 months, mean change (SD)		*P* value		6 months, mean (SD)		6 months, mean change (SD)		*P* value
Weight (lb)				198.73 (40.15)		190.54 (40.54)		−8.19 (7.51)		<.001		189.01 (41.47)		−9.72 (10.78)		<.001
BMI (kg/m^2^)				31.77 (5.02)		30.41 (5.2)		−1.36 (1.25)		<.001		30.16 (5.35)		−1.61 (1.76)		<.001
Hunger				56.37 (16.69)		45.28 (21.79)		−11.1 (21.66)		<.001		43.34 (19.85)		−13.02 (22.23)		<.001
**Food Craving Inventory**																
	Sweets		2.53 (0.81)		2.2 (0.77)		−0.33 (0.62)		<.001		2.07 (0.73)		−0.46 (0.63)		<.001	
	High fats		1.88 (0.65)		1.62 (0.54)		−0.26 (0.52)		<.001		1.57 (0.56)		−0.31 (0.53)		<.001	
	Carbohydrates and starches		2.25 (0.73)		1.9 (0.61)		−0.35 (0.6)		<.001		1.79 (0.61)		−0.45 (0.57)		<.001	
	Fast foods		2.8 (0.81)		2.44 (0.7)		−0.36 (0.7)		<.001		2.31 (0.70)		−0.49 (0.77)		<.001	
	Total score		2.3 (0.59)		1.98 (0.53)		−0.32 (0.51)		<.001		1.88 (0.53)		−0.42 (0.52)		<.001	
**Fruit and vegetable intake**																
		Fruit	0.83 (0.77)		1.19 (1.06)		0.36 (0.8)		<.001		1.01 (1.02)		0.18 (0.82)		.007	
		Salad	0.43 (0.45)		0.72 (0.71)		0.29 (0.56)		<.001		0.62 (0.62)		0.18 (0.53)		<.001	
		Other vegetables	0.79 (0.66)		1.04 (0.85)		0.25 (0.76)		<.001		0.94 (0.82)		0.16 (0.70)		.006	
Total physical activity (MET^a^ min/wk)				2285.88 (3772.17)		3156.58 (5242.86)		870.69 (4396.63)		.02		3069.83 (4054.51)		783.95 (3611.88)		.008
Sedentary behavior (MET min/d)				491.77 (211.47)		423 (191.93)		−68.77 (159.19)		<.001		407.68 (192.53)		−84.10 (169.31)		<.001
Total Pittsburgh Sleep Quality Index				6.2 (2.89)		5.98 (3.39)		−0.22 (2.76)		.34		5.64 (3.29)		−0.56 (2.56)		.008
Total impact of weight on quality of life				74.93 (15.64)		80.18 (14.78)		5.26 (10.65)		<.001		81.55 (15.20)		6.63 (11.51)		<.001
Total self-compassion				3.3 (0.67)		3.34 (0.69)		0.05 (0.57)		.32		3.38 (0.70)		0.09 (0.56)		.06
Total World Health Organization-5 Well-Being Index				58.93 (17.99)		64.03 (18.5)		5.1 (14.7)		<.001		64.89 (18.23)		5.96 (15.36)		<.001

^a^MET: metabolic equivalent.

### Participation

The mean percentage of workshops attended was 50.88% (SD 30.39%) during the first 3 months and 40.33% (SD 29.83%) over 6 months (Table 3). The mean percentage of wellness check-ins attended was 61.59% (SD 31.97%) in the first 3 months and 49.62% (SD 31.79%) over 6 months. The mean percentage of weeks in which participants attended either a workshop or a wellness check-in was 70.75% (SD 31.67%) in the first 3 months and 56.71% (SD 33.71%) over 6 months. The mean percentage of weeks in which participants engaged in the private community (ie, posts, comments and replies, and reactions) was 34.92% (SD 36.67%) in the first 3 months and 25.13% (SD 29.29%) over 6 months. The mean percentage of weeks in which participants attended a workshop, a wellness check-in, or engaged in the private group was 74.75% (SD 31.33%) in the first 3 months and 60.46% (SD 33.83%) over 6 months (Table 3). In terms of WW app use, participants logged in on a mean of 68.61% (SD 32.19%) of the days in the first 3 months and 56.02% (SD 32.99%) of the days over 6 months. When examining participation in any of the intervention components (workshops, wellness check-ins, private group, and mobile app), the average percentage of weeks of participation was 87.53% (SD 23.40%) in the first 3 months and 77.67% (SD 28.69%) over 6 months. Overall, 59.5% (91/153) of the sample participated in ≥1 of the intervention components on ≥80% of the weeks of the program.

Participants tracked food on a mean of 53.62% (SD 33.48%) of the days from baseline to 3 months and 39.12% (SD 30.76%) of the days from baseline to 6 months. The mean percentage of days in which participants tracked physical activity was 66.95% (SD 42.63%) at 3 months and 63.31% (SD 43.59%) at 6 months. On average, participants tracked weight on 24.22% (SD 20.28%) of the days from baseline to 3 months and 19.32% (SD 18.24%) of the days from baseline to 6 months.

**Table 3:** Participation in workshops, wellness check-ins, private group, and mobile app (n=153).

Participation	3 months, mean (SD)	6 months, mean (SD)
Weekly workshops attended	6.11 (3.65)	9.68 (7.16)
Percentage of workshops attended	50.88 (30.39)	40.33 (29.83)
Weekly wellness check-ins attended	7.39 (3.84)	11.91 (7.63)
Percentage of wellness check-ins attended	61.59 (31.97)	49.62 (31.79)
Weeks engaged in private group	4.19 (4.40)	6.03 (7.03)
Percentage of weeks engaged in private group	34.92 (36.67)	25.13 (29.29)
Weekly app log-ins	10.37 (2.91)	18.12 (7.01)
Percentage of weekly app log-ins	86.44 (24.28)	75.52 (29.23)
Weeks attended a workshop or wellness check-in or engaged in private group or app log-ins	10.50 (2.81)	18.64 (6.89)
Percentage of weeks attended a workshop or wellness check-in or engaged in private group or app log-ins	87.53 (23.40)	77.67 (28.69)

### Satisfaction

Of the 140 participants who completed the 6-month follow-up, 116 (82.9%) agreed or strongly agreed that the new WW program made them more mindful of their choices (ie, food, activity, mindset, and sleep), 102 (72.9%) agreed or strongly agreed that the program made them feel healthier, 96 (68.6%) agreed or strongly agreed that the program made healthy living simple, and 95 (67.9%) agreed or strongly agreed that the program helped them to lower their risk of health conditions (such as diabetes or high blood pressure). Furthermore, 67.9% (95/140) of the participants agreed or strongly agreed that the WW app helped them to stay on track, and 67.9% (95/140) agreed or strongly agreed that the WW app simplified weight loss. Of the 140 participants, 95 (67.9%) agreed or strongly agreed that the workshops were welcoming and that they felt part of the group, and 95 (67.9%) agreed or strongly agreed that the tools and information provided in the program were relevant to people who were similar to them (Table 4).

**Table 4:** Participants’ satisfaction and feedback on the new WeightWatchers (WW) program (n=140).

Satisfaction and feedback			Values, n (%)
**The new** **WW** **program...**			
	Makes me more mindful of my choices (i.e., food, activity, mindset, and sleep).	116 (82.9)	
	Makes me feel healthier.	102 (72.9)	
	Makes healthy living simple.	96 (68.6)	
	Helps lower my risk of health conditions (like diabetes or high blood pressure).	95 (67.9)	
	Is the most flexible weight loss program I’ve tried.	95 (67.9)	
	Helps me lose weight AND improve other areas of my life.	94 (67.1)	
	Empowers me to lose weight without missing out on life.	92 (65.7)	
	Allows me to eat the foods I love and still lose weight.	89 (63.6)	
	Is individualized for me/is made just for me and my life.	84 (60)	
**The** **WW** **app...**			
	Helps me to stay on track.	95 (67.9)	
	Helps simplify weight loss.	95 (67.9)	
**General satisfaction and feedback**			
	The workshops were welcoming and I felt part of the group.	95 (67.9)	
	The tools and information provided in the program are relevant to people who are similar to me.	95 (67.9)	
	Following the program allowed me to still participate in cultural and/or religious practices.	93 (66.4)	

### Behavioral Measures

#### Hunger

There was a significant decrease in hunger from baseline to 3 months (mean change −11.1, SD 21.66 and mean percentage change −11.64%, SD 63.26%) and from baseline to 6 months (mean change −13.02, SD 22.23 and mean percentage change −12.84%, SD 62.79%; all *P* values <.001).

#### Food Craving

From baseline to 3 months, there were significant decreases in craving for sweets (mean change −0.33, SD 0.62), high fats (mean change −0.26, SD 0.52), carbohydrates or starches (mean change −0.35, SD 0.6), fast foods (mean change −0.36, SD 0.7), and total food craving (mean change −0.32, SD 0.51; all *P* values <.001). From baseline to 6 months, we also observed significant decreases in craving for sweets (mean change −0.46, SD 0.63), high fats (mean change −0.31, SD 0.53), carbohydrates or starches (mean change −0.45, SD 0.57), fast foods (mean change −0.49, SD 0.77), and total food craving (mean change −0.42, SD 0.52; all *P* values <.001; Table 2).

#### Fruit and Vegetable Intake

Fruit intake increased significantly at 3 months (mean change 0.36, SD 0.8; *P<*.001) and 6 months (mean change 0.18, SD 0.82; *P*=.007). Salad intake increased significantly at both 3 months (mean change 0.29, SD 0.56; *P*<.001) and 6 months (mean change 0.18, SD 0.53; *P*<.001). Vegetable intake increased significantly among participants at 3 months (mean change 0.25, SD 0.76; *P*<.001) and 6 months (mean change 0.16, SD 0.70; *P*=.006; Table 2).

#### Physical Activity, Sedentary Behavior, and Sleep

Participants’ total physical activity increased significantly at 3 months (mean change 870.69, SD 4396.63 MET min/wk; *P*=.02) and 6 months (mean change 783.95, SD 3611.88 MET min/wk; *P*=.008). Sedentary behavior also significantly decreased at 3 months (mean change −68.77, SD 159.19 min/d; *P*<.001) and 6 months (mean change −84.10, SD 169.31 min/d; *P*<.001; Table 2). We observed a significant improvement in sleep quality at 6 months (mean change −0.56, SD 2.56; *P*=.008) but not at 3 months (Table 2).

#### Psychosocial Outcomes

The total impact of weight on quality of life significantly improved at both 3 months (mean change 5.26, SD 10.65; *P*<.001) and 6 months (mean change 6.63, SD 11.51, *P*<.001). We did not observe a significant change in self-compassion at 3 or 6 months. Participants’ well-being increased significantly at 3 months (mean change 5.1, SD 14.7; *P*<.001) and 6 months (mean change 5.96, SD 15.36; *P*<.001; Table 2). As hypothesized, positive behavioral automaticity significantly increased at both 3 months (mean change 0.74, SD 0.94; *P*<.001) and 6 months (mean change 0.94, SD 1.69; *P*<.001). As hypothesized, negative behavioral automaticity significantly decreased at both 3 months (mean change −0.74, SD 1.44; *P*<.001) and 6 months (mean change −0.61, SD 1.57; *P*<.001).

## Discussion

### Principal Findings

The findings revealed that a digital personalized WW program delivered under real-world conditions (meaning that it was delivered as it would be commercially such that participants were not reminded to attend meetings or followed up with if they missed meetings, as is often the case in weight loss trials) resulted in a mean weight loss of nearly 10 (SD 10.78) lb or 5.05% (SD 5.59%) of the baseline weight at 6 months, with 51% (78/153) of the sample losing ≥5%. A recent meta-analysis of 10 digital weight loss interventions revealed a mean weight loss of 5.3 (range 2.86-13.7) lb [1], which places our findings at the higher end of the range for digital programs. In this study, participants had numerous ways to participate, including attendance at workshops and wellness check-ins, participation in a private virtual group, and logging in to the app. The mean percentage of weeks in which participants took part in ≥1 of the intervention components was 87.53% (SD 23.40%) at 3 months and 77.67% (SD 28.69%) at 6 months, and 59.5% (91/153) of the sample took part in ≥1 of the intervention components on ≥80% of the weeks of the program. This suggests that generally most of the participants (91/153, 59.5%) were engaged in ≥1 aspects of the program throughout the 6 months. The majority of participants expressed satisfaction with the program, agreeing that the program made them feel healthier (102/140, 72.9%), made them more mindful of their choices (116/140, 82.9%), and made healthy living simple (96/140, 68.6%).

Previous trials of personalized weight loss interventions have focused personalization on feedback [28,29], educational materials, and other intervention content [5]. This study adds to the literature by personalizing the dietary goal via the PersonalPoints allowance and ZPFs in such a way that allows flexibility in the diet. In this trial, 153 participants used 121 different variations of the weight management program plan. Dynamic personalization in previous studies has typically been carried out at static intervals, such as daily or weekly [5], whereas the WW PersonalPoints program allows participants to decide whether and when they want to change the inputs. Future research is needed to compare the impact of participant-initiated versus static interval–driven dynamic personalization on weight loss and participant preferences and to better assess which inputs and targets of personalization lead to the best outcomes.

The PersonalPoints program was designed to encourage healthy diet choices, including intake of fruit, vegetables, and legumes, all of which have positive impacts on appetite and satiety [30]. Indeed, we observed increases in fruit and vegetable intake and reductions in hunger and craving at both 3 and 6 months. One might assume that reducing intake from less healthy foods such as sweets, starches, and fast foods might increase the desire for such foods; but, on the contrary, we observed significant decreases in cravings for these foods. This is consistent with literature that shows that long-term restriction of specific foods results in reduced cravings for these foods [31]. Traditional diet-tracking apps that focus on calorie tracking treat all calories the same, but, ultimately, the goal for chronic disease prevention is to help people change the landscape of their diet in a way that maximizes diet quality while minimizing food cravings and hunger. Future studies should explore whether greater use of ZPFs by participants is associated with more reduction in hunger and food cravings in the WW program. One challenge to assessing the impact of the use of ZPFs is that, by definition, the user is not required to track them. This makes it difficult to know to what extent the participants used ZPFs and the ways in which they used the ZPFs to reduce the burden of self-monitoring and to manage hunger and cravings.

We also observed effects of the personalized program on physical activity, sedentary behavior, and sleep. The WW program allows users to add extra weekly PersonalPoints for the exercise they perform, with higher-intensity forms of exercise adding more points, which may motivate participants to exercise. Time spent in sedentary activities declined by an average of nearly 90 minutes per day, which is further evidence that the program nudged people toward more active lifestyles. WW describes *4 pillars*—food, activity, sleep, and mindset—as the foundation of this program, and each is addressed in all aspects of the program. We also observed a significant improvement in sleep quality, which is evidence that the sleep-related content in the program had an impact on participants.

The mindset component, the fourth pillar of the WW program, encompasses techniques from cognitive behavioral therapy and positive psychology to help users cope with setbacks, approach the process of weight loss with self-compassion, and stay focused on lifestyle changes that have a positive impact on overall well-being and happiness [32]. We found significant improvements in well-being and weight-related quality of life, which revealed that participants were indeed receptive to learning these concepts and incorporating them into their weight loss journey. However, we did not find a significant effect on self-compassion. Future research should examine ways to improve self-compassion in the context of weight management. Studies show that people with obesity are often the target of weight stigma [33] and that internalized weight stigma, which is when individuals apply stigmatizing beliefs about their body size to themselves, can lead to worse weight loss outcomes [34]. For this reason, weight loss programs should address how people think about their weight, how they deal with setbacks during a weight loss attempt, and how to deal with internalized weight stigma. Future studies should examine the additive effect of digitally delivered self-compassion–based intervention content on weight loss outcomes.

This study can be considered in the context of trials of previous versions of the WW program. Tate et al [35] conducted a 1-arm trial of the WW food program that used the SmartPoints system and weekly in-person workshops. In the intervention, all participants received the same list of ZPFs. The findings revealed that 60% of the participants lost ≥5% of their baseline weight at 6 months compared with 51% in this trial. We cannot draw definitive conclusions regarding the reasons for greater observed weight loss in their trial compared with ours; however, in the trial conducted by Tate et al [35], 86.8% (132/152) of the participants were former WW members compared with 25.5% (39/153) in our trial. We found that former WW members tended to lose more weight than participants who were naive to WW, with 59% (23/39) of the former losing ≥5% of their baseline weight at 6 months compared with 47.7% (52/109) of the latter. Of the 153 participants, 5 (3.3%) were excluded from this analysis because they did not indicate whether they had used a WW program before. People who have experience with the WW program and are willing to use it again may have better success rates than people who are new to the program, which could explain the greater weight loss in the trial conducted by Tate et al [35]. Interestingly, Tate et al [35] found that being a former member of WW was not a predictor of losing 10% of baseline weight at 6 months, although with only 13% of the sample not having had used the WW program in the past, it is not clear whether they had the power to find an effect [35]. Trials testing commercial programs might produce more representative findings if they recruit individuals who do not already have experience and familiarity with the program. A trial by Marrero et al [36] that tested the efficacy of the WW program in 2013-2014 included the mobile app and weekly in-person workshops in adults with prediabetes and a BMI value in the overweight or obese range. They reported mean percentage weight change from baseline to 6 months of 5.53%, which is comparable with the finding in our trial (5.05%) but less than that in the trial by Tate et al [35] (7.89%). The trial by Marrero et al [36] did not report the proportion of participants who had previously been WW members, making it difficult to discern whether this could have possibly played a role in the differences across trials. Thomas et al [37] conducted a randomized trial involving 12 months of free access to the WW PointsPlus program that could be accessed by mobile app or PC. In that trial, participants were not provided weekly workshops. The findings revealed that 24.5% of the participants lost ≥5% of their baseline weight at 3 months and 25.5% lost ≥5% of their baseline weight at 12 months. Weight loss was not reported at 6 months. Our trial, the trial by Marrero et al [36], and the trial by Tate et al [35] provided weekly workshops, which may explain the higher rates of weight loss observed compared with the weight loss rates observed in the trial by Thomas et al [37]. Future studies should compare outcomes from the WW program with weekly workshops versus without and with virtual workshops versus in-person workshops, as well as differential effects by whether participants had previous experience with WW.

Workshop attendance varied across our trial as well as the 2 aforementioned trials using WW workshops [35,36]. In our trial, on average, participants attended 40% (9.6/24) of the weekly virtual workshops, whereas in the trial by Tate et al [35], on average, participants attended 70.5% (16.91/24) of the in-person workshops. In the trial by Marrero et al [36], the mean number of weekly in-person workshops attended was 42% (21.6/52). In our trial, in addition to the weekly workshops, participants were offered weekly individual check-ins with a coach, and they participated in an average of 50% of these check-ins. We also offered participation in a web-based community, and participants engaged on an average of 25% of the weeks over 6 months. Taking into account all 3 ways in which participants could interact with coaches in our intervention, on average, participants engaged in ≥1 of these ways on 60.46% of the weeks of the program. As different studies provide different ways to participate and likely vary in how they manage participation (eg, sending reminders and following up with participants who do not participate), it is difficult to speculate on the reasons for varying participation across studies. Our trial and the trial by Marrero et al [36] had similar weight losses and participation rates, both of which were somewhat lower than those in the trial by Tate et al [35]. Future studies should examine the impact of participation-enhancing methods that are feasible to implement in the real world.

This study has limitations to consider. First, this was a single-arm trial, which means that the absence of a comparison group prevents comparisons with no treatment or other programs. That said, no-treatment control groups in weight loss trials produce negligible weight loss (ie, 0.2 lb) [38]. Second, our sample consisted largely of female participants (107/153, 69.9%), not unlike most weight loss trials [39], but this reduces our ability to generalize the findings to male participants, especially male participants from a minoritized racial or ethnic group. However, 42.5% (65/153) of our trial participants identified as non-White, which is higher than the percentage in many previous trials of various versions of the WW program and other studied behavioral weight management programs [35,40,41]. This is a strength in light of systematic reviews revealing underrepresentation of racial and ethnic minority groups in lifestyle interventions [42,43]. Finally, we are unable to isolate the impact of any particular personalization piece or intervention component. Future studies should test the differential effects of personalization components, individual check-ins, and a members-only digital community group on weight loss outcomes. An opportunity for future exploration is to assess whether the percentage of days in which participants were adherent to the daily PersonalPoints budget was associated with greater weight loss.

### Conclusions

In conclusion, this personalized, fully digital, and scalable WW program, which emphasized 4 pillars (food, activity, sleep, and mindset) and leveraged >300 ZPFs, personalized ZPF lists, and the PersonalPoints plan, resulted in statistically and clinically significant weight loss in half (78/153, 51%) of the participants. PersonalPoints and ZPFs are features designed to simplify the task of dietary self-monitoring while nudging people toward a high-quality diet. WW first offered this program to the public in November 2021. Future studies should examine how different forms of personalization in digital weight loss programs affect weight loss outcomes.

## Appendix

Multimedia Appendix 1 WeightWatchers app screenshots.

Multimedia Appendix 2 In-app questions assessing food preferences and physical activity for the PersonalPoints engine.

Multimedia Appendix 3 Weekly workshop topics.

## Abbreviations

GPAQ: Global Physical Activity Questionnaire
MET: metabolic equivalent
REDCap: Research Electronic Data Capture
WW: WeightWatchers
ZPF: ZeroPoint food
