# Supplementary figures and images for: An Evaluation of a Personalized Multicomponent Commercial Digital Weight Management Program: Single-Arm Behavioral Trial
Source: J Med Internet Res. 2023 Aug 29;25:e44955. doi: 10.2196/44955 (PMC10498321; doi:10.2196/44955)

Multimedia Appendix 1. WW App Screenshots

**
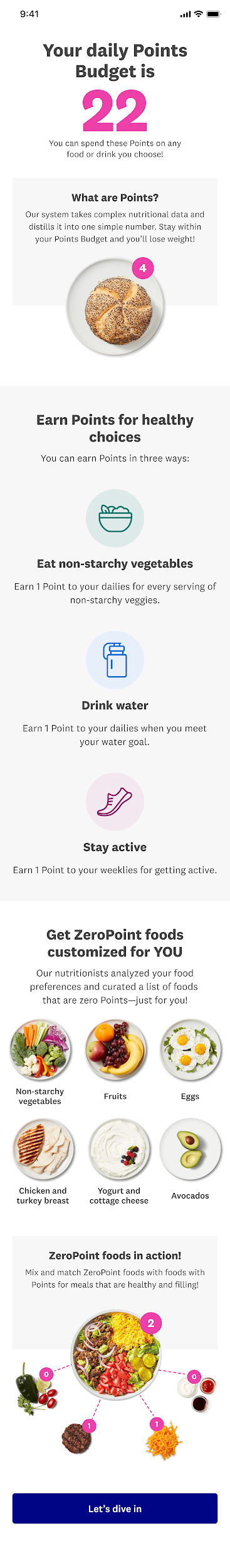

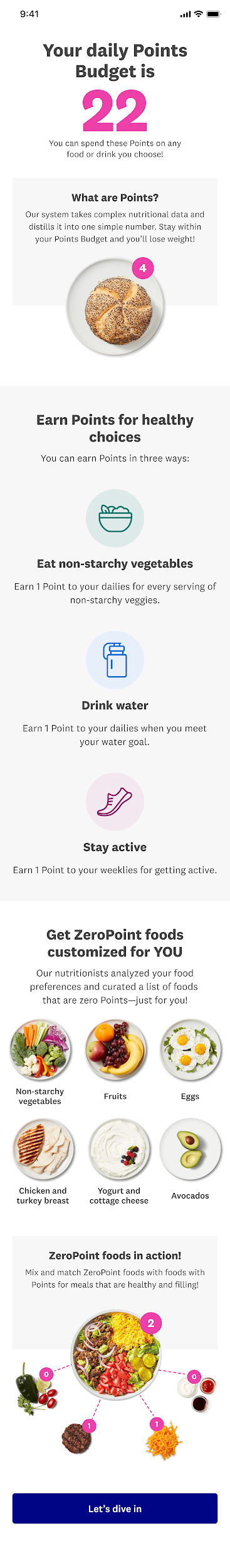
**


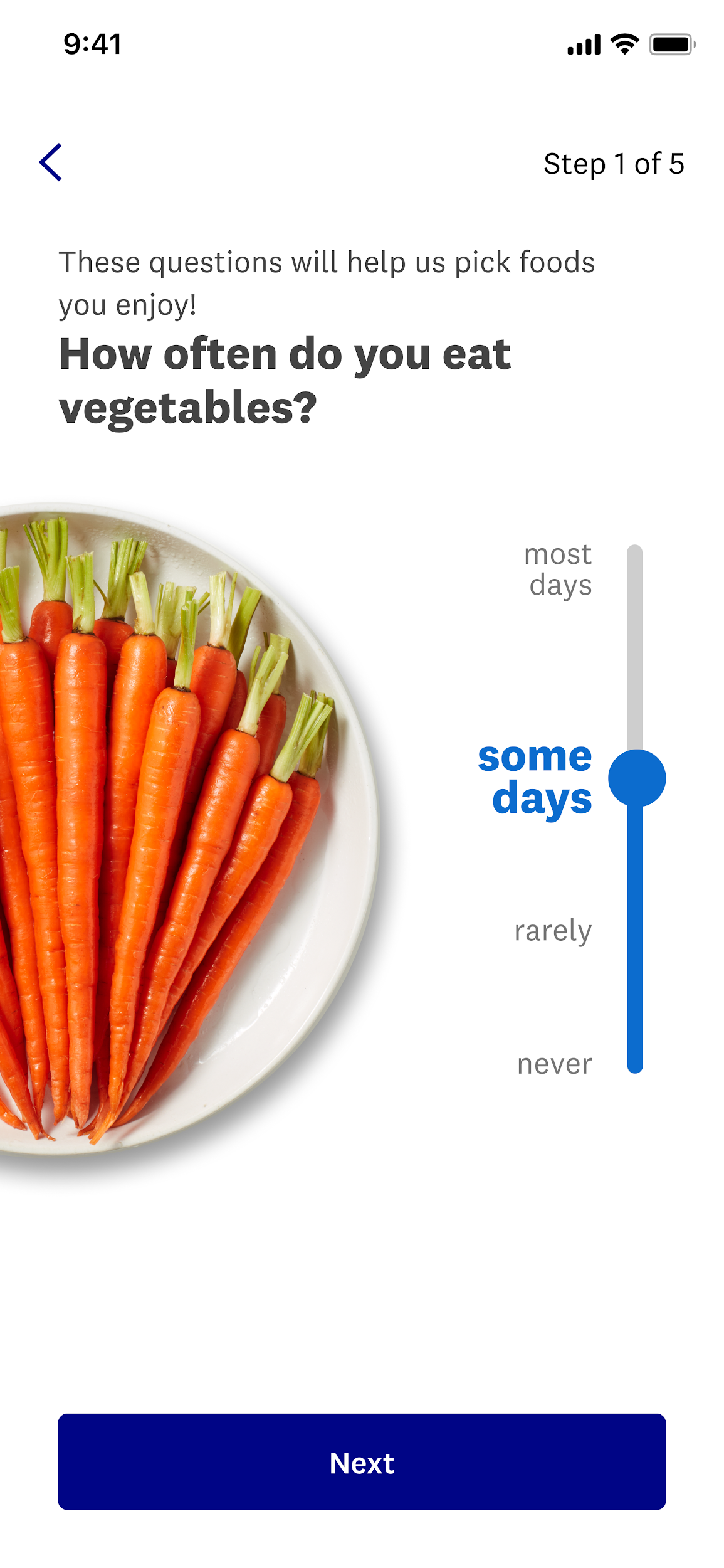

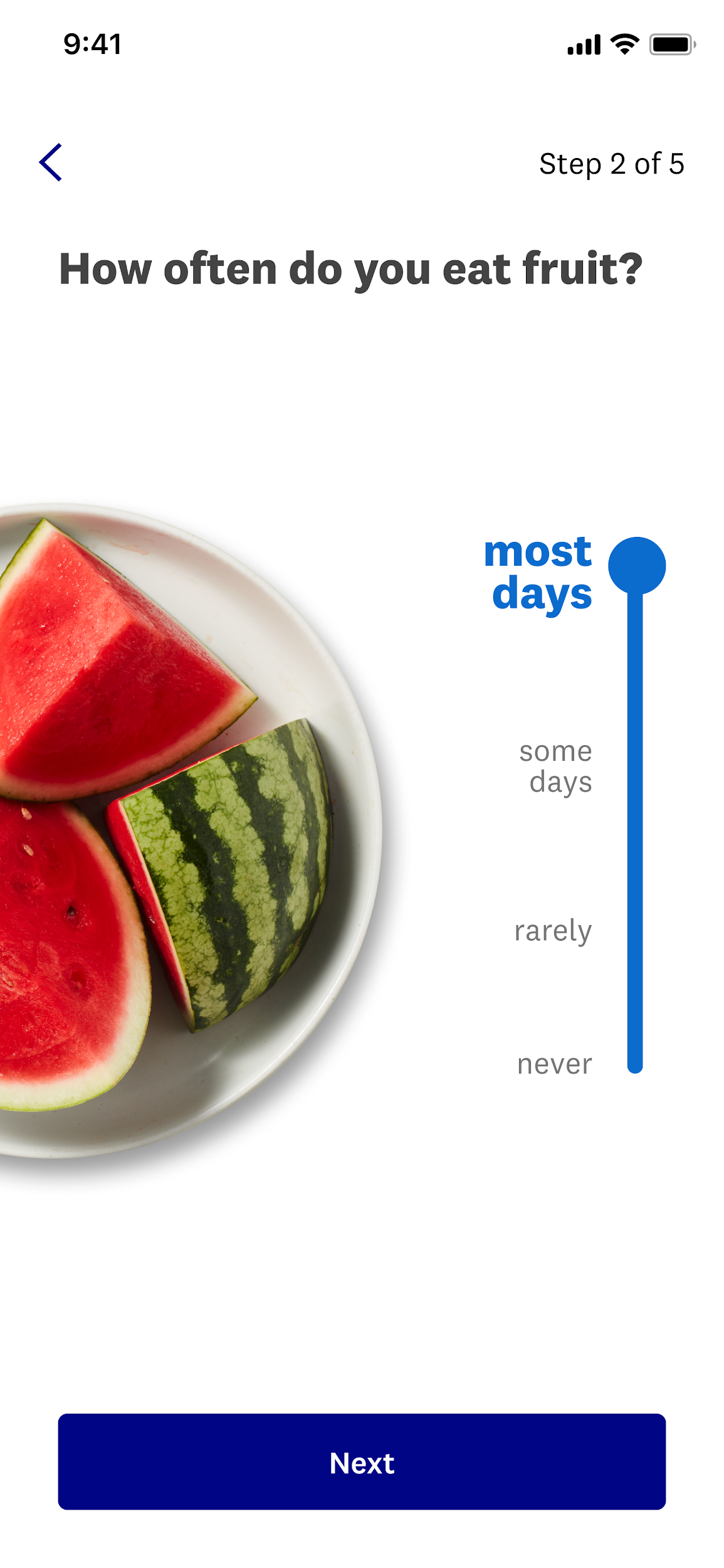

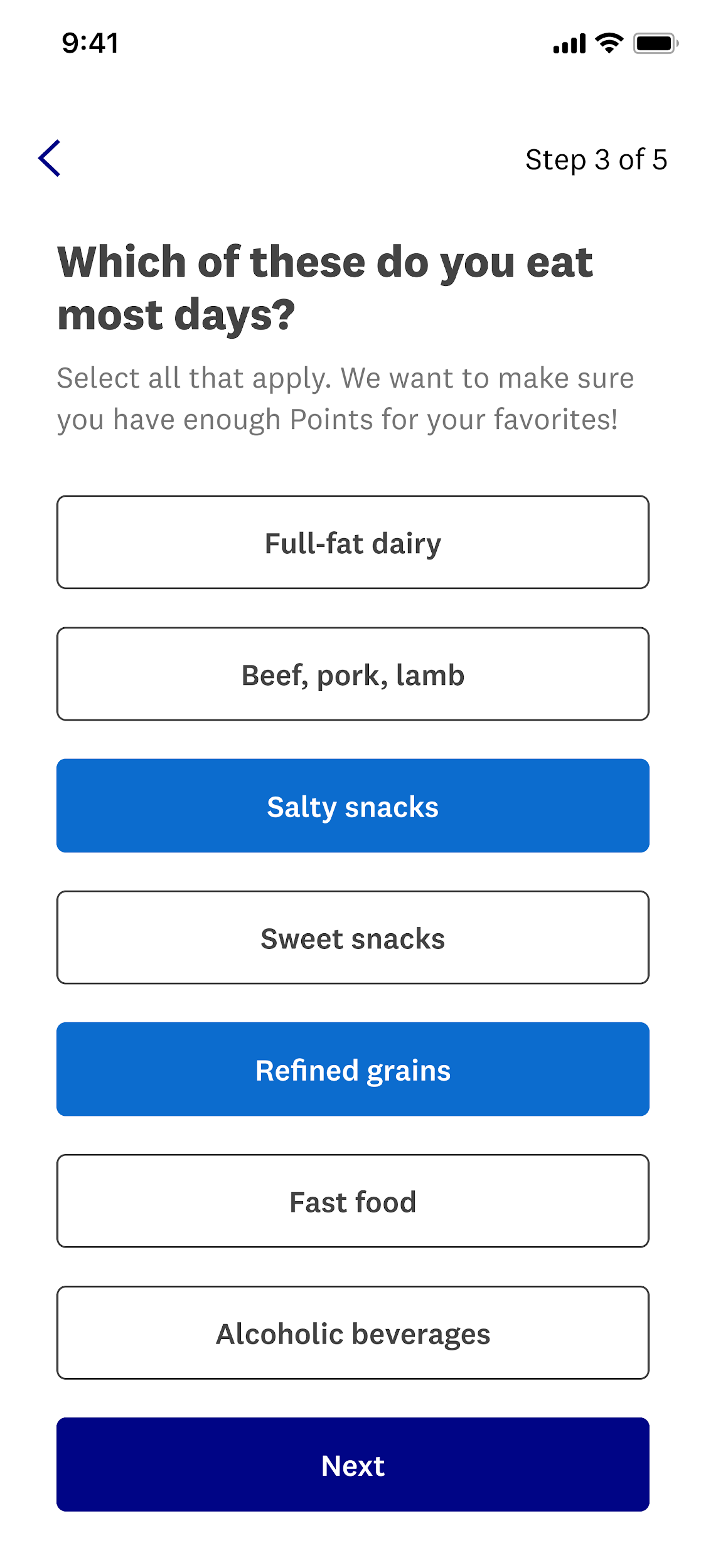

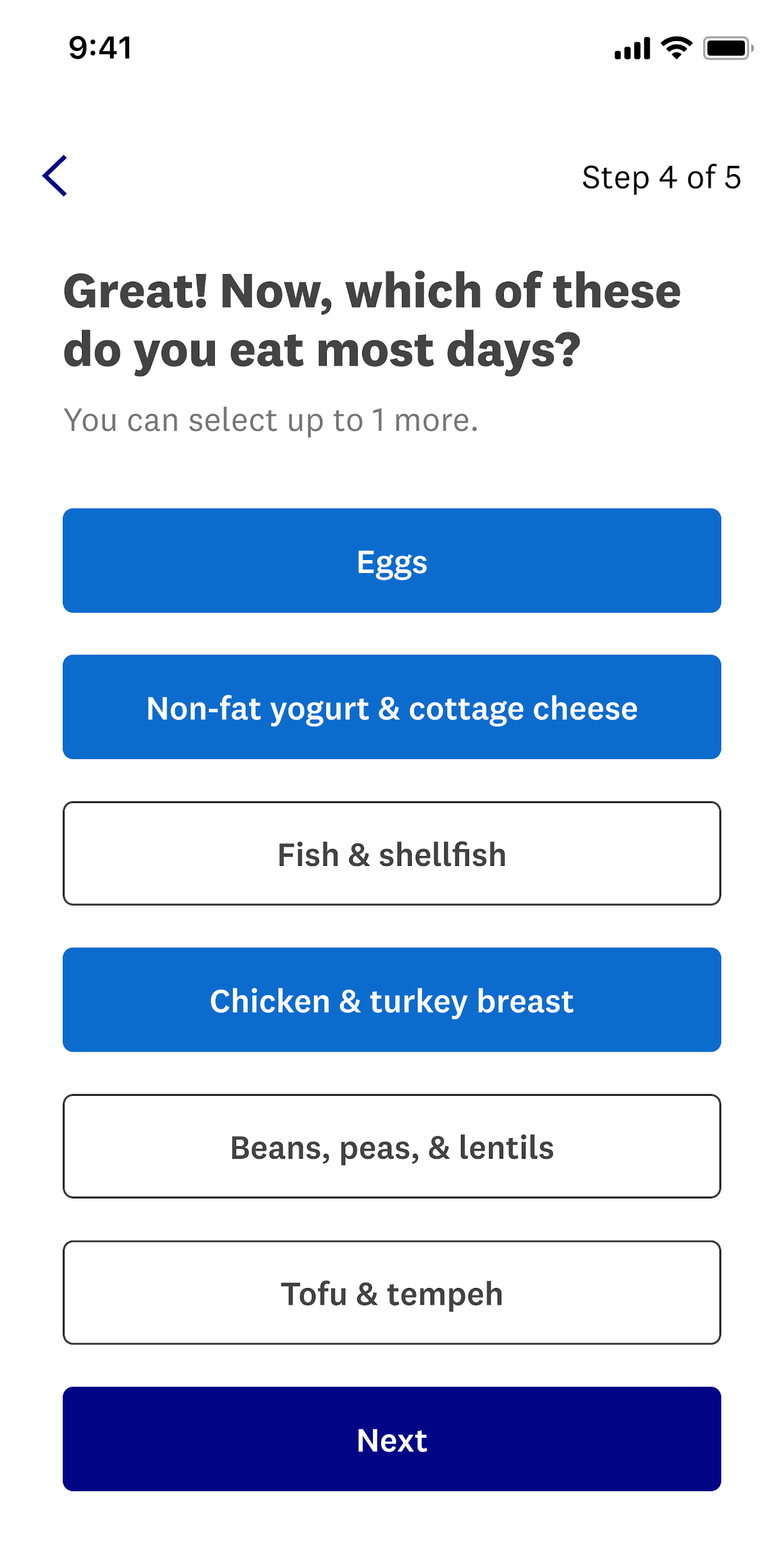

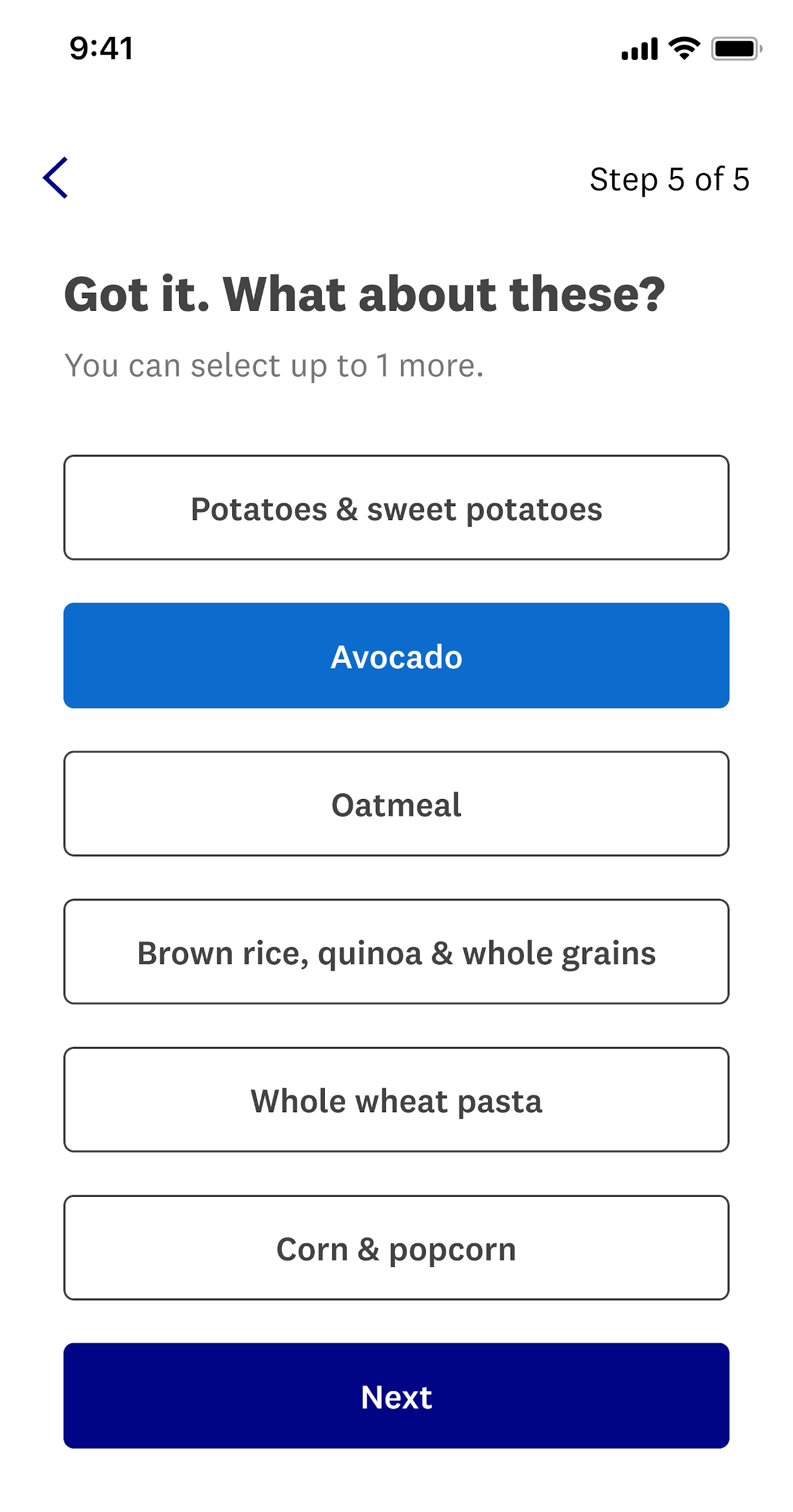

Supplement: Multimedia Appendix 1 [file jmir_v25i1e44955_app1.docx]
