# Supplementary material for: An Evaluation of a Personalized Multicomponent Commercial Digital Weight Management Program: Single-Arm Behavioral Trial
Source: J Med Internet Res. 2023 Aug 29;25:e44955. doi: 10.2196/44955 (PMC10498321; doi:10.2196/44955)
Supplement: Multimedia Appendix 2 [file jmir_v25i1e44955_app2.docx]

**Multimedia Appendix 2. In App Questions Assessing Food Preferences and Physical Activity for the PersonalPoint Engine**

| **Food preference questions** |
| --- |
| **How often do you enjoy eating vegetables?** [Most days, some days, rarely, never] |
| **How often do you enjoy eating fruit?** [Most days, some days, rarely, never] |
| **Which of these do you enjoy eating most days?** [*Select all that apply*: full-fat dairy (milk, cream,  cheese, yogurt), beef/pork/lamb, salty snacks, sweet snacks, refined grains (bread, cracked, cereals),  fast food (burgers, french fries, pizza, sandwiches), alcoholic beverages] |
| **Which of these protein-rich foods do you enjoy most days?** [*Max number of selections based on*  *answers to previous questions, must select at least 1*: eggs, non-fat yogurt & cottage cheese (plain,  unsweetened), fish & shellfish, chicken & turkey breast (skinless), beans/peas/lentils, tofu & tempeh] |
| **Which of these do you enjoy eating most days?** [*Max number of selections based on answers to*  *previous questions*: potatoes & sweet potatoes, oatmeal, whole wheat pasta, brown rice & quinoa,  avocado, corn & popcorn (plain, air-popped), none of these) |
| **Physical Activity Questions** |
| **For your activity target, which of these would you like to use?** [Steps, Minutes, I’m not sure] |
| **If steps is chosen:**  **On average, how many steps do you take each day, Monday - Friday?**  [Don’t know or <3,000 steps, 3,000-3,999, 4,000-4,999, 5,000-5,999, 6,000-6,999, 7,000-7,999,  8,000-8,999, 9,000-9,999, 10,000+]  **On average, how many steps do you take each day over the weekend?** [Don’t know or <3,000  steps, 3,000-3,999; 4,000-4,999, 5,000-5,999, 6,000-6,999, 7,000-7,999, 8,000-8,999, 9,000-  9,999, 10,000+] |
| **If minutes is chosen:**  **On average, how many days are you active from Monday to Friday?** [0, 1, 2, 3, 4, 5]  *[If 1 or more days was selected***]**  **On average, how many minutes are you active on each of those days, Monday to Friday?**  [<10. 20, 30, 40, 50, 60+]  **How many days are you active on the weekend?** [0, 1, 2]  *[If 1 or more days was selected]* **On average, how many minutes are you active on each day**  **over the weekend?** [<10, 20, 30, 40, 50, 60+] |
| **If Not Sure is chosen:**  **Do you own a step-tracking device, like a FitBit, Amazon Halo, or Apple Watch?** [Yes, No]  **[***If no step-tracking device***]** Member is sent into the question flow for minutes.  **[***If yes to own a step-tracking device***] Do you want to use it to track your activity?** [Yes, No]  **[***If yes to wanting to use it to track activity***]** Member is sent into the question flow for steps.  **[***If no to wanting to use it to track activity***]** Member is sent into the question flow for minutes. |
