# Supplementary material for: An Evaluation of a Personalized Multicomponent Commercial Digital Weight Management Program: Single-Arm Behavioral Trial
Source: J Med Internet Res. 2023 Aug 29;25:e44955. doi: 10.2196/44955 (PMC10498321; doi:10.2196/44955)
Supplement: Multimedia Appendix 3 [file jmir_v25i1e44955_app3.docx]

Multimedia Appendix 3. Weekly Workshop Topics

| **Week** | **Topic** |
| --- | --- |
| 1 | **Intro to WW program** |
| 2 | **Intro to ZeroPoint foods** |
| 3 | **Tracking** Food, Activity, and Sleep |
| 4 | **Setting STAR Goals**- Specific, Truly Doable, Active, Relevant |
| 5 | **Break up sitting** - Make the behavior you want to do easier by adding friction to the unwanted behavior |
| 6 | **Reflect on non-scale victories**- Identify things that have changed other than the number on the scale and celebrate those |
| 7 | **Identify your "go to"s** - Find your go-to lower point foods that you enjoy and can always have on hand |
| 8 | **Bedtime routine**-Set up a routine to help you relax and fall asleep |
| 9 | **Remember strengths**- Identify your strengths and create a visible affirmation that helps you reflect daily |
| 10 | **Temptation bundling**- Pairing something you love with something you “should” or “must” do that you don’t enjoy |
| 11 | **Monitor, Modify or Maintain (Habits)**- Catch yourself in a habit, think about how it happened, decide if you want to change or keep it. |
| 12 | **Reinforcement**- Choose a new healthy habit with an immediate positive impact so it's easier to maintain. |
| 13 | **Choose your cues**- Add a new habit to something you already consistently do |
| 14 | **Micro-Habits & Habit Stacking**- Break up a new habit you want to form in to tiny, easy steps |
| 15 | **Strengthen your brain**- Identify your current paths and write new ones that bring you closer to your long term goals. |
| 16 | **Strengthen your body**- Find a strength training activity that works for you |
| 17 | **Meal planning for strength**- Plan meals in advance to include options that boost muscle and bone strength |
| 18 | **Pathways to self-compassion**- Create a self-compassion affirmation to use during setbacks or challenges |
| 19 | **Set a goal your future self can actually do**- Break down goals to help intention become action |
| 20 | **Switch up your eating routine**- Build a meal around a new ingredient or method to keep things fresh |
| 21 | **Set up your bedroom environment**- Make your bedroom more sleep friendly |
| 22 | **Focus on what you can control**- Learn to identify what you can control, you can influence, and what you can’t control. |
| 23 | **Make time for what you enjoy**- Prioritize things you find fulfilling |
| 24 | **Reflection & Next Steps-** Reflect on the past 6 months and create an action plan |
